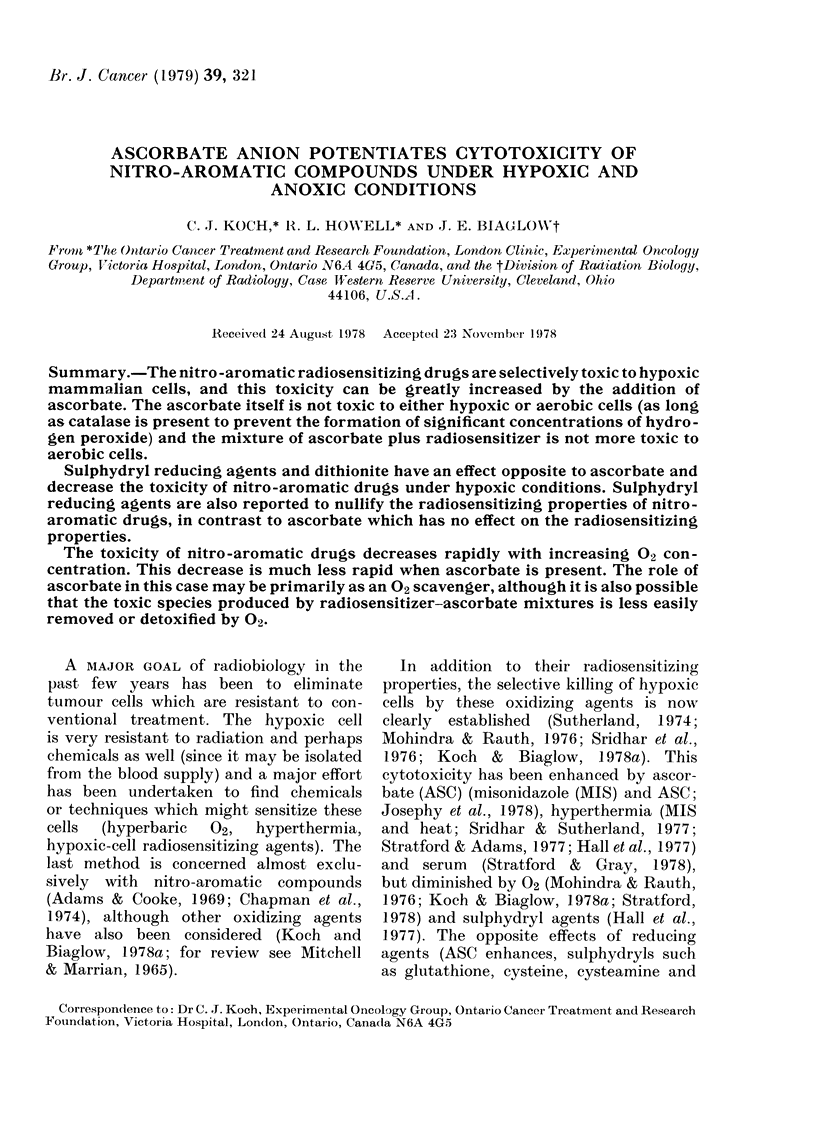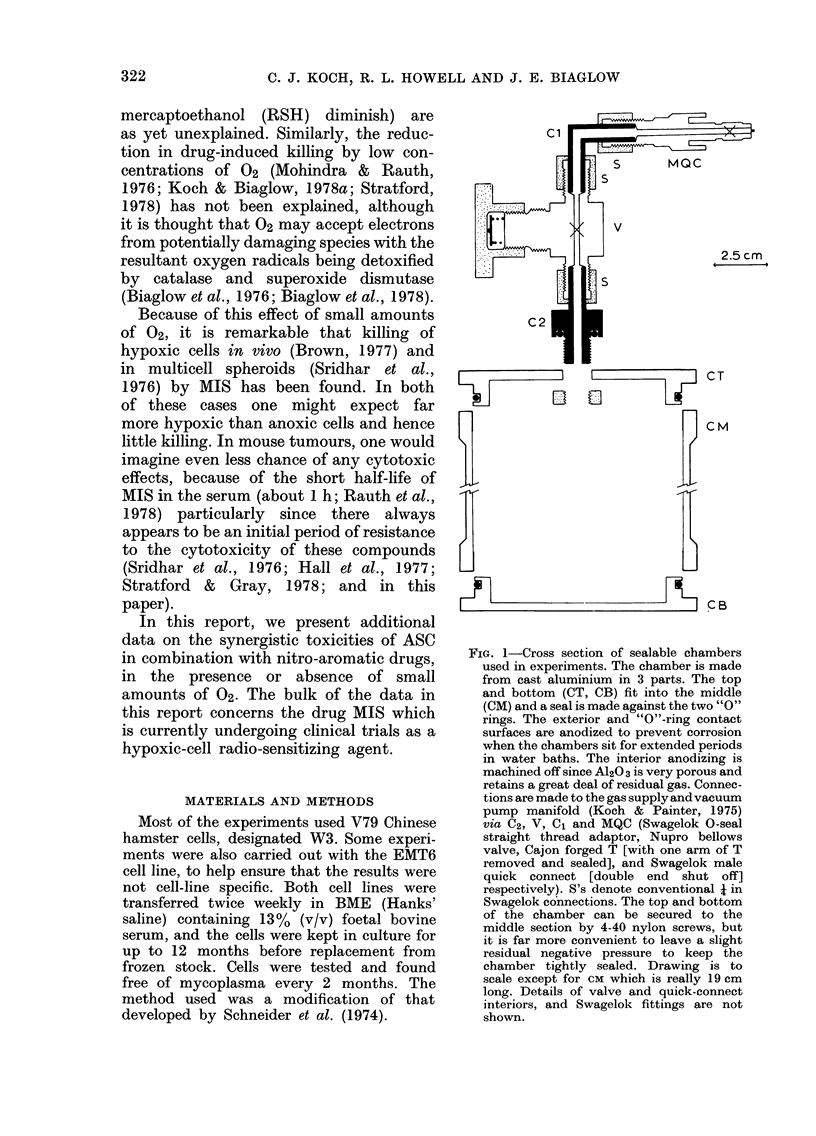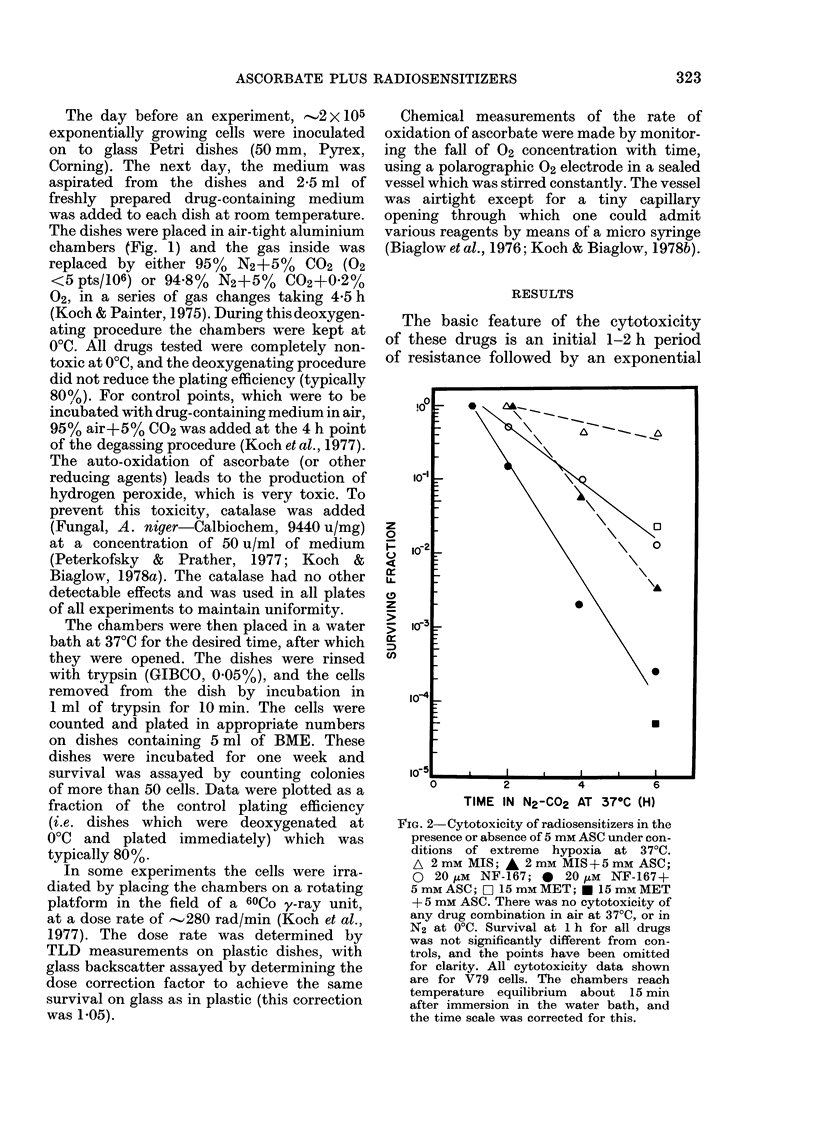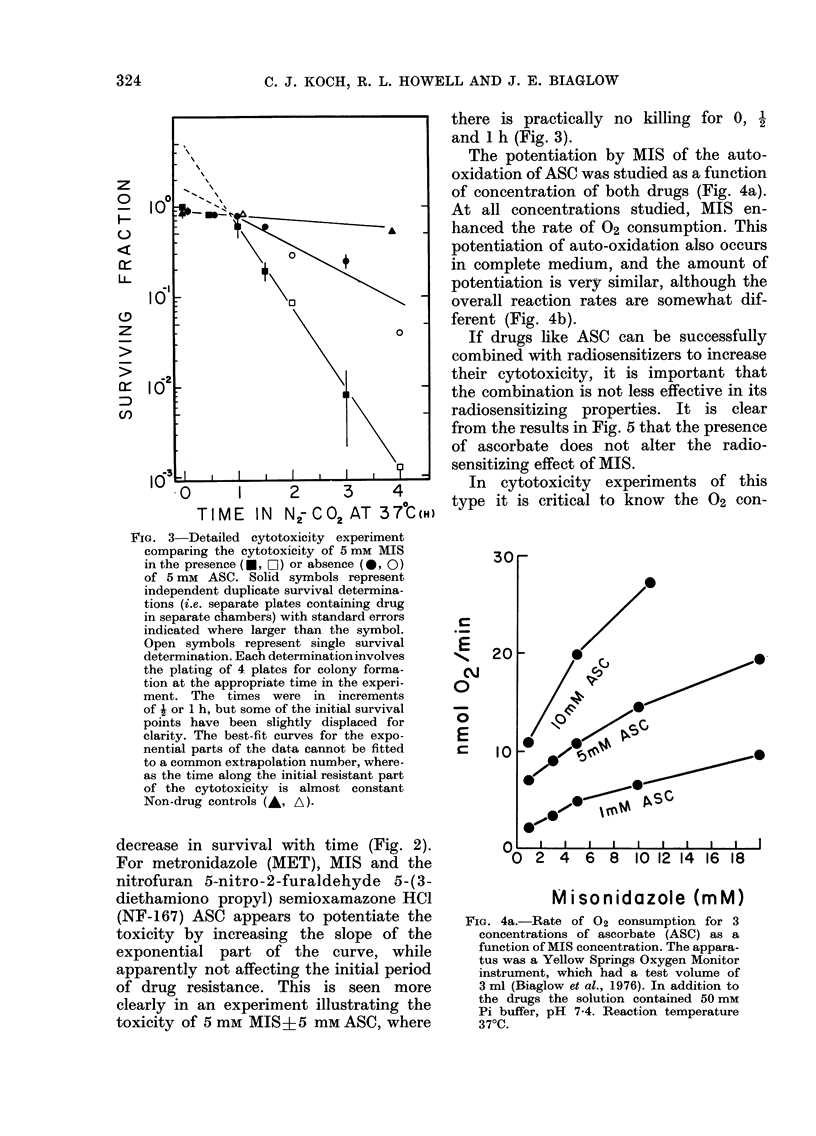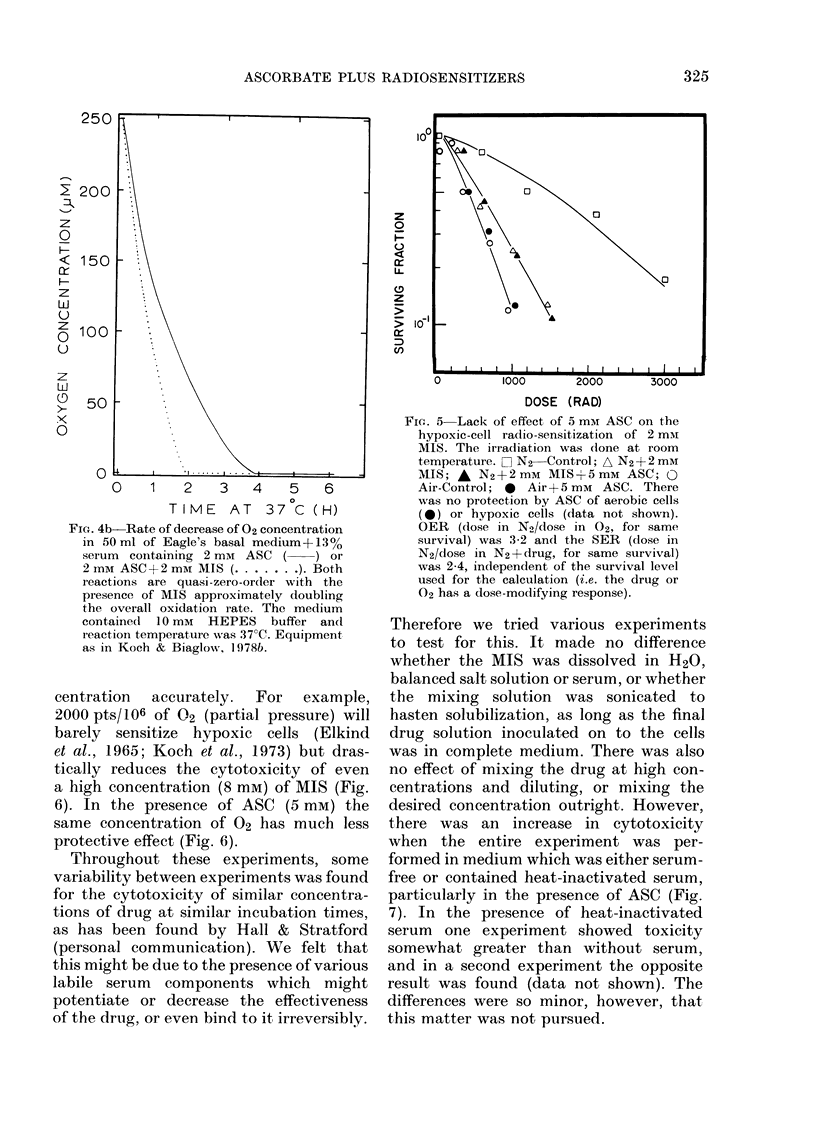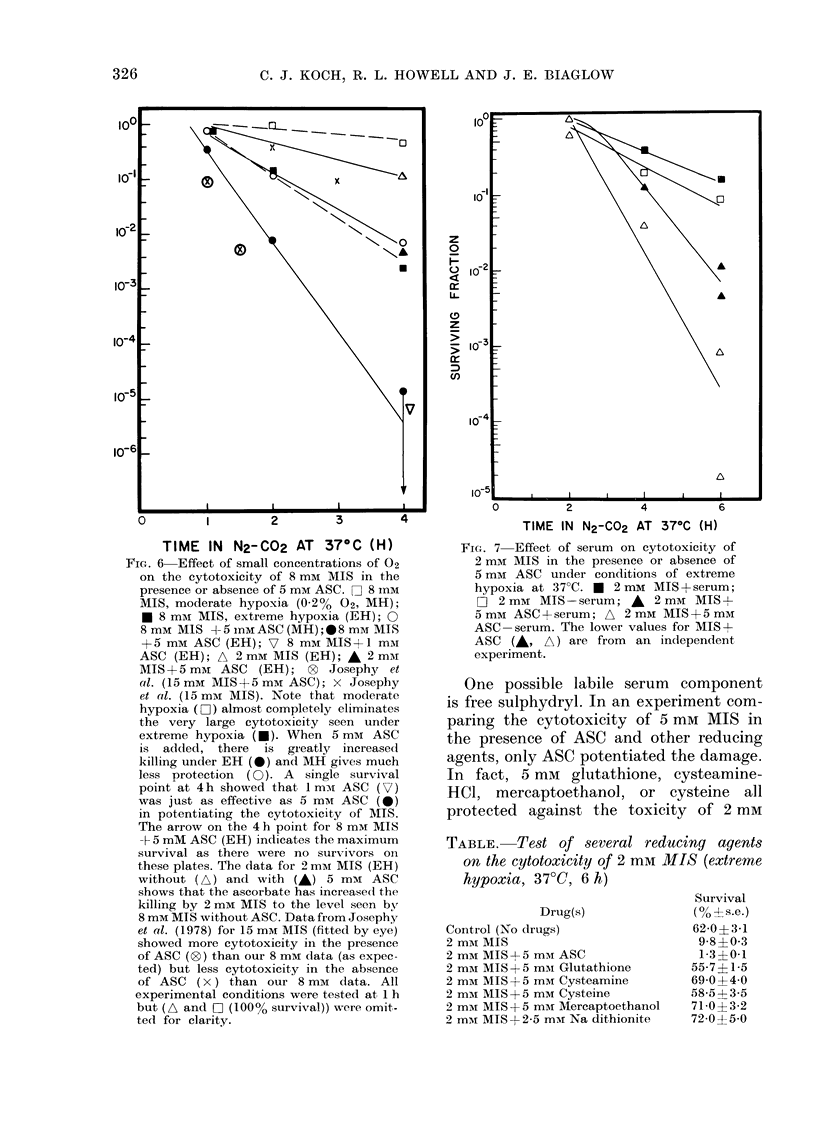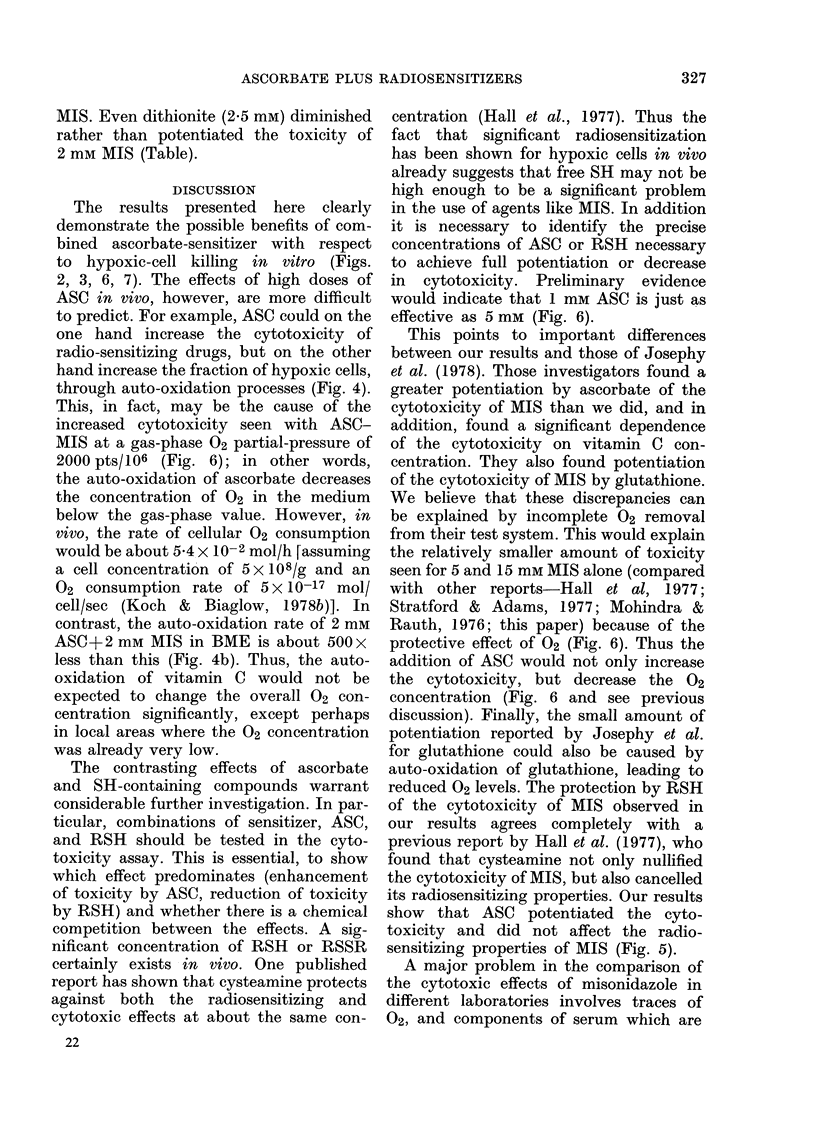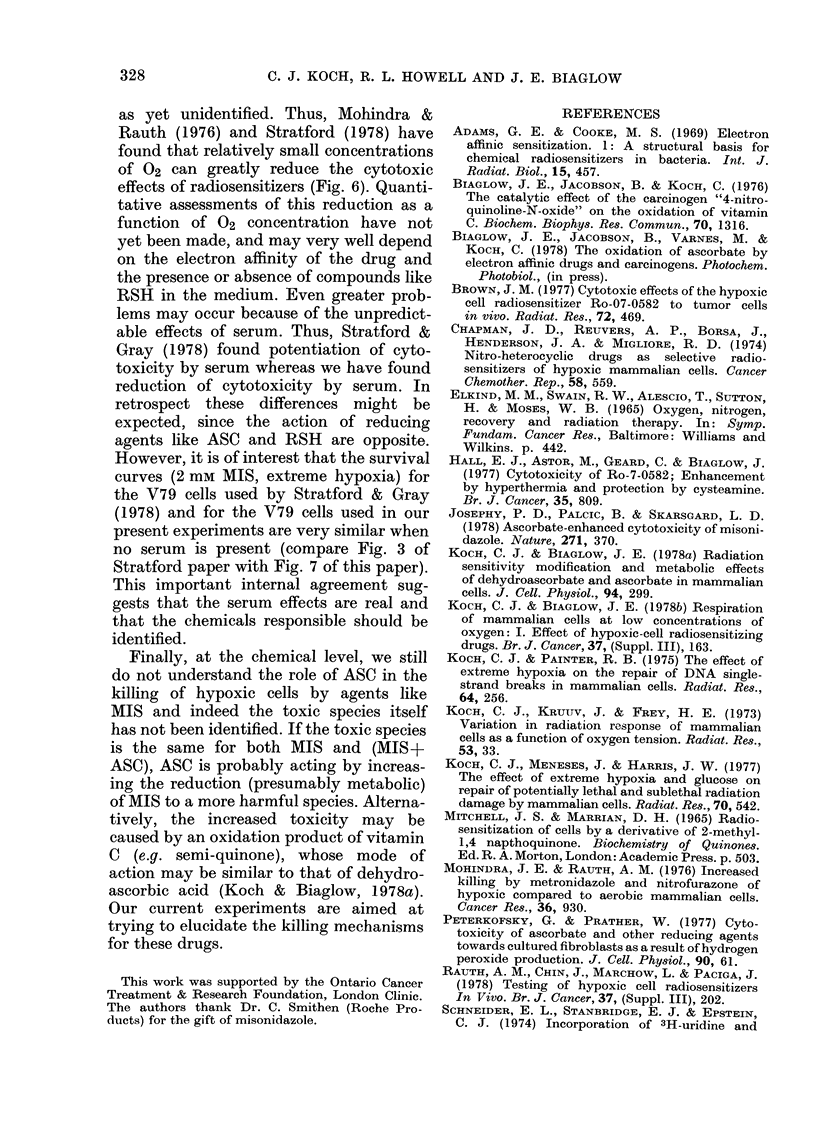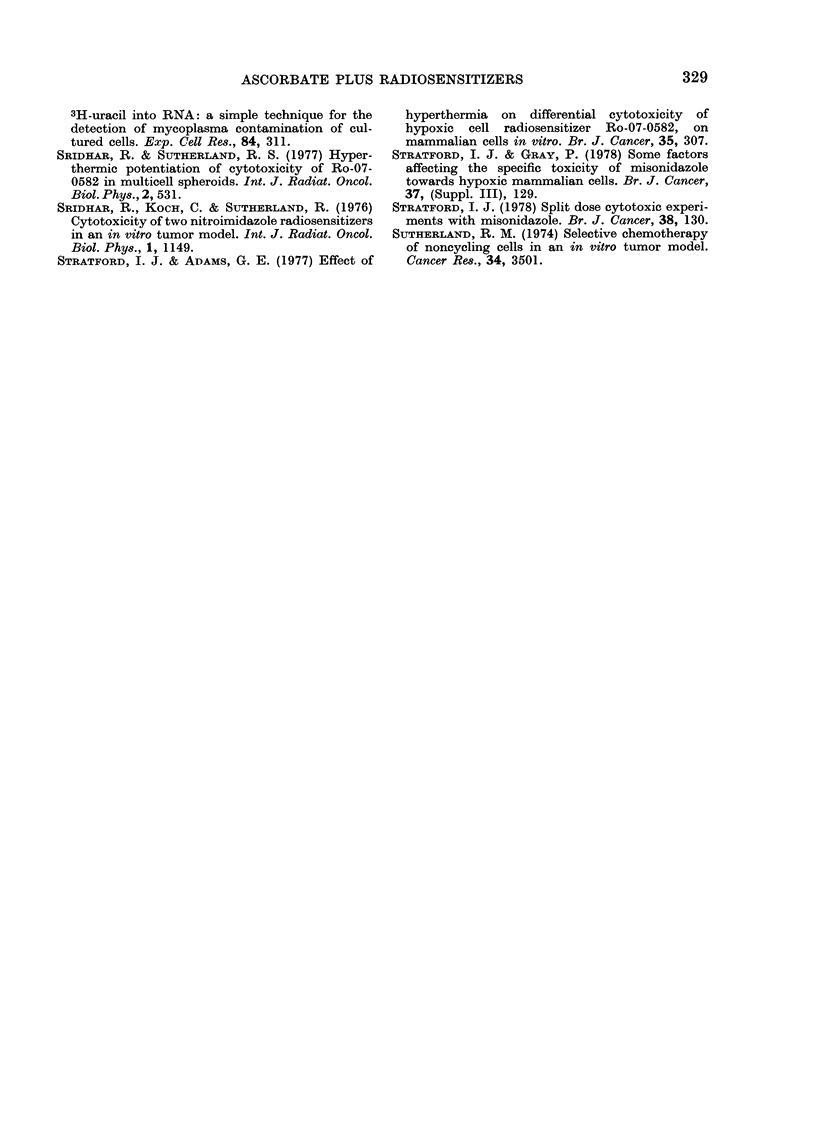# Ascorbate anion potentiates cytotoxicity of nitro-aromatic compounds under hypoxic and anoxic conditions.

**DOI:** 10.1038/bjc.1979.56

**Published:** 1979-03

**Authors:** C. J. Koch, R. L. Howell, J. E. Biaglow

## Abstract

The nitro-aromatic radiosensitizing drugs are selectively toxic to hypoxic mammalian cells, and this toxicity can be greatly increased by the addition of ascorbate. The ascorbate itself is not toxic to either hypoxic or aerobic cells (as long as catalase is present to prevent the formation of significant concentrations of hydrogen peroxide) and the mixture of ascorbate plus radiosensitizer is not more toxic to aerobic cells. Sulphydryl reducing agents and dithionite have an effect opposite to ascorbate and decrease the toxicity of nitro-aromatic drugs under hypoxic conditions. Sulphydryl reducing agents are also reported to nullify the radiosensitizing properties of nitro-aromatic drugs, in contrast to ascorbate which has no effect on the radiosensitizing properties. The toxicity of nitro-aromatic drugs decreases rapidly with increasing O2 concentration. This decrease is much less rapid when ascorbate is present. The role of ascorbate in this case may be primarily as an O2 scavenger, although it is also possible that the toxic species produced by radiosensitizer-ascorbate mixtures is less easily removed or detoxified by O2.


*Br. J. Cancer* (1979) **39**, 321

### ASCORBATE ANION POTENTIATES CYTOTOXICITY OF NITRO-AROMATIC COMPOUNDS UNDER HYPOXIC AND ANOXIC CONDITIONS

C. J. KOCH,* R. L. HOWELL* AND J. E. BIAGLOW†

*From *The Ontario Cancer Treatment and Research Foundation, London Clinic, Experimental Oncology Group, Victoria Hospital, London, Ontario N6A 4G5, Canada, and the †Division of Radiation Biology, Department of Radiology, Case Western Reserve University, Cleveland, Ohio 44106, U.S.A.*

Received 24 August 1978 Accepted 23 November 1978

**Summary.—The nitro-aromatic radiosensitizing drugs are selectively toxic to hypoxic mammalian cells, and this toxicity can be greatly increased by the addition of ascorbate. The ascorbate itself is not toxic to either hypoxic or aerobic cells (as long as catalase is present to prevent the formation of significant concentrations of hydrogen peroxide) and the mixture of ascorbate plus radiosensitizer is not more toxic to aerobic cells.**

**Sulphydryl reducing agents and dithionite have an effect opposite to ascorbate and decrease the toxicity of nitro-aromatic drugs under hypoxic conditions. Sulphydryl reducing agents are also reported to nullify the radiosensitizing properties of nitro-aromatic drugs, in contrast to ascorbate which has no effect on the radiosensitizing properties.**

**The toxicity of nitro-aromatic drugs decreases rapidly with increasing $O_2$ concentration. This decrease is much less rapid when ascorbate is present. The role of ascorbate in this case may be primarily as an $O_2$ scavenger, although it is also possible that the toxic species produced by radiosensitizer–ascorbate mixtures is less easily removed or detoxified by $O_2$.**

A MAJOR GOAL of radiobiology in the past few years has been to eliminate tumour cells which are resistant to conventional treatment. The hypoxic cell is very resistant to radiation and perhaps chemicals as well (since it may be isolated from the blood supply) and a major effort has been undertaken to find chemicals or techniques which might sensitize these cells (hyperbaric $O_2$, hyperthermia, hypoxic-cell radiosensitizing agents). The last method is concerned almost exclusively with nitro-aromatic compounds (Adams & Cooke, 1969; Chapman *et al.*, 1974), although other oxidizing agents have also been considered (Koch and Biaglow, 1978*a*; for review see Mitchell & Marrian, 1965).

In addition to their radiosensitizing properties, the selective killing of hypoxic cells by these oxidizing agents is now clearly established (Sutherland, 1974; Mohindra & Rauth, 1976; Sridhar *et al.*, 1976; Koch & Biaglow, 1978*a*). This cytotoxicity has been enhanced by ascorbate (ASC) (misonidazole (MIS) and ASC; Josephy *et al.*, 1978), hyperthermia (MIS and heat; Sridhar & Sutherland, 1977; Stratford & Adams, 1977; Hall *et al.*, 1977) and serum (Stratford & Gray, 1978), but diminished by $O_2$ (Mohindra & Rauth, 1976; Koch & Biaglow, 1978*a*; Stratford, 1978) and sulphydryl agents (Hall *et al.*, 1977). The opposite effects of reducing agents (ASC enhances, sulphydryls such as glutathione, cysteine, cysteamine and

Correspondence to: Dr C. J. Koch, Experimental Oncology Group, Ontario Cancer Treatment and Research Foundation, Victoria Hospital, London, Ontario, Canada N6A 4G5

mercaptoethanol (RSH) diminish) are as yet unexplained. Similarly, the reduction in drug-induced killing by low concentrations of $O_2$ (Mohindra & Rauth, 1976; Koch & Biaglow, 1978*a*; Stratford, 1978) has not been explained, although it is thought that $O_2$ may accept electrons from potentially damaging species with the resultant oxygen radicals being detoxified by catalase and superoxide dismutase (Biaglow *et al.*, 1976; Biaglow *et al.*, 1978).

Because of this effect of small amounts of $O_2$, it is remarkable that killing of hypoxic cells *in vivo* (Brown, 1977) and in multicell spheroids (Sridhar *et al.*, 1976) by MIS has been found. In both of these cases one might expect far more hypoxic than anoxic cells and hence little killing. In mouse tumours, one would imagine even less chance of any cytotoxic effects, because of the short half-life of MIS in the serum (about 1 h; Rauth *et al.*, 1978) particularly since there always appears to be an initial period of resistance to the cytotoxicity of these compounds (Sridhar *et al.*, 1976; Hall *et al.*, 1977; Stratford & Gray, 1978; and in this paper).

In this report, we present additional data on the synergistic toxicities of ASC in combination with nitro-aromatic drugs, in the presence or absence of small amounts of $O_2$. The bulk of the data in this report concerns the drug MIS which is currently undergoing clinical trials as a hypoxic-cell radio-sensitizing agent.

FIG. 1—Cross section of sealable chambers used in experiments. The chamber is made from cast aluminium in 3 parts. The top and bottom (CT, CB) fit into the middle (CM) and a seal is made against the two "O" rings. The exterior and "O"-ring contact surfaces are anodized to prevent corrosion when the chambers sit for extended periods in water baths. The interior anodizing is machined off since $Al_2O_3$ is very porous and retains a great deal of residual gas. Connections are made to the gas supply and vacuum pump manifold (Koch & Painter, 1975) *via* $C_2$, V, $C_1$ and MQC (Swagelok O-seal straight thread adaptor, Nupro bellows valve, Cajon forged T [with one arm of T removed and sealed], and Swagelok male quick connect [double end shut off] respectively). S's denote conventional ¼ in Swagelok connections. The top and bottom of the chamber can be secured to the middle section by 4-40 nylon screws, but it is far more convenient to leave a slight residual negative pressure to keep the chamber tightly sealed. Drawing is to scale except for CM which is really 19 cm long. Details of valve and quick-connect interiors, and Swagelok fittings are not shown.

### MATERIALS AND METHODS

Most of the experiments used V79 Chinese hamster cells, designated W3. Some experiments were also carried out with the EMT6 cell line, to help ensure that the results were not cell-line specific. Both cell lines were transferred twice weekly in BME (Hanks' saline) containing 13% (v/v) foetal bovine serum, and the cells were kept in culture for up to 12 months before replacement from frozen stock. Cells were tested and found free of mycoplasma every 2 months. The method used was a modification of that developed by Schneider *et al.* (1974).

The day before an experiment, ~$2 \times 10^5$ exponentially growing cells were inoculated on to glass Petri dishes (50 mm, Pyrex, Corning). The next day, the medium was aspirated from the dishes and 2·5 ml of freshly prepared drug-containing medium was added to each dish at room temperature. The dishes were placed in air-tight aluminium chambers (Fig. 1) and the gas inside was replaced by either 95% $N_2$+5% $CO_2$ ($O_2$ <5 pts/$10^6$) or 94·8% $N_2$+5% $CO_2$+0·2% $O_2$, in a series of gas changes taking 4·5 h (Koch & Painter, 1975). During this deoxygenating procedure the chambers were kept at 0°C. All drugs tested were completely non-toxic at 0°C, and the deoxygenating procedure did not reduce the plating efficiency (typically 80%). For control points, which were to be incubated with drug-containing medium in air, 95% air+5% $CO_2$ was added at the 4 h point of the degassing procedure (Koch *et al.*, 1977). The auto-oxidation of ascorbate (or other reducing agents) leads to the production of hydrogen peroxide, which is very toxic. To prevent this toxicity, catalase was added (Fungal, *A. niger*—Calbiochem, 9440 u/mg) at a concentration of 50 u/ml of medium (Peterkofsky & Prather, 1977; Koch & Biaglow, 1978*a*). The catalase had no other detectable effects and was used in all plates of all experiments to maintain uniformity.

The chambers were then placed in a water bath at 37°C for the desired time, after which they were opened. The dishes were rinsed with trypsin (GIBCO, 0·05%), and the cells removed from the dish by incubation in 1 ml of trypsin for 10 min. The cells were counted and plated in appropriate numbers on dishes containing 5 ml of BME. These dishes were incubated for one week and survival was assayed by counting colonies of more than 50 cells. Data were plotted as a fraction of the control plating efficiency (*i.e.* dishes which were deoxygenated at 0°C and plated immediately) which was typically 80%.

In some experiments the cells were irradiated by placing the chambers on a rotating platform in the field of a $^{60}$Co $\gamma$-ray unit, at a dose rate of ~280 rad/min (Koch *et al.*, 1977). The dose rate was determined by TLD measurements on plastic dishes, with glass backscatter assayed by determining the dose correction factor to achieve the same survival on glass as in plastic (this correction was 1·05).

Chemical measurements of the rate of oxidation of ascorbate were made by monitoring the fall of $O_2$ concentration with time, using a polarographic $O_2$ electrode in a sealed vessel which was stirred constantly. The vessel was airtight except for a tiny capillary opening through which one could admit various reagents by means of a micro syringe (Biaglow *et al.*, 1976; Koch & Biaglow, 1978*b*).

### RESULTS

The basic feature of the cytotoxicity of these drugs is an initial 1–2 h period of resistance followed by an exponential

FIG. 2—Cytotoxicity of radiosensitizers in the presence or absence of 5 mM ASC under conditions of extreme hypoxia at 37°C. △ 2 mM MIS; ▲ 2 mM MIS+5 mM ASC; ○ 20 $\mu$M NF-167; ● 20 $\mu$M NF-167+5 mM ASC; □ 15 mM MET; ■ 15 mM MET +5 mM ASC. There was no cytotoxicity of any drug combination in air at 37°C, or in $N_2$ at 0°C. Survival at 1 h for all drugs was not significantly different from controls, and the points have been omitted for clarity. All cytotoxicity data shown are for V79 cells. The chambers reach temperature equilibrium about 15 min after immersion in the water bath, and the time scale was corrected for this.

FIG. 3—Detailed cytotoxicity experiment comparing the cytotoxicity of 5 mM MIS in the presence (■, □) or absence (●, ○) of 5 mM ASC. Solid symbols represent independent duplicate survival determinations (*i.e.* separate plates containing drug in separate chambers) with standard errors indicated where larger than the symbol. Open symbols represent single survival determination. Each determination involves the plating of 4 plates for colony formation at the appropriate time in the experiment. The times were in increments of ½ or 1 h, but some of the initial survival points have been slightly displaced for clarity. The best-fit curves for the exponential parts of the data cannot be fitted to a common extrapolation number, whereas the time along the initial resistant part of the cytotoxicity is almost constant Non-drug controls (▲, △).

decrease in survival with time (Fig. 2). For metronidazole (MET), MIS and the nitrofuran 5-nitro-2-furaldehyde 5-(3-diethamiono propyl) semioxamazone HCl (NF-167) ASC appears to potentiate the toxicity by increasing the slope of the exponential part of the curve, while apparently not affecting the initial period of drug resistance. This is seen more clearly in an experiment illustrating the toxicity of 5 mM MIS+5 mM ASC, where there is practically no killing for 0, ½ and 1 h (Fig. 3).

The potentiation by MIS of the auto-oxidation of ASC was studied as a function of concentration of both drugs (Fig. 4a). At all concentrations studied, MIS enhanced the rate of $O_2$ consumption. This potentiation of auto-oxidation also occurs in complete medium, and the amount of potentiation is very similar, although the overall reaction rates are somewhat different (Fig. 4b).

If drugs like ASC can be successfully combined with radiosensitizers to increase their cytotoxicity, it is important that the combination is not less effective in its radiosensitizing properties. It is clear from the results in Fig. 5 that the presence of ascorbate does not alter the radiosensitizing effect of MIS.

In cytotoxicity experiments of this type it is critical to know the $O_2$ con-

FIG. 4a.—Rate of $O_2$ consumption for 3 concentrations of ascorbate (ASC) as a function of MIS concentration. The apparatus was a Yellow Springs Oxygen Monitor instrument, which had a test volume of 3 ml (Biaglow *et al.*, 1976). In addition to the drugs the solution contained 50 mM Pi buffer, pH 7·4. Reaction temperature 37°C.

FIG. 4b—Rate of decrease of $O_2$ concentration in 50 ml of Eagle's basal medium+13% serum containing 2 mM ASC (——) or 2 mM ASC+2 mM MIS (. . . . . . .). Both reactions are quasi-zero-order with the presence of MIS approximately doubling the overall oxidation rate. The medium contained 10 mM HEPES buffer and reaction temperature was 37°C. Equipment as in Koch & Biaglow, 1978*b*.

FIG. 5—Lack of effect of 5 mM ASC on the hypoxic-cell radio-sensitization of 2 mM MIS. The irradiation was done at room temperature. □ $N_2$—Control; △ $N_2$+2 mM MIS; ▲ $N_2$+2 mM MIS+5 mM ASC; ○ Air-Control; ● Air+5 mM ASC. There was no protection by ASC of aerobic cells (●) or hypoxic cells (data not shown). OER (dose in $N_2$/dose in $O_2$, for same survival) was 3·2 and the SER (dose in $N_2$/dose in $N_2$+drug, for same survival) was 2·4, independent of the survival level used for the calculation (*i.e.* the drug or $O_2$ has a dose-modifying response).

centration accurately. For example, 2000 pts/$10^6$ of $O_2$ (partial pressure) will barely sensitize hypoxic cells (Elkind *et al.*, 1965; Koch *et al.*, 1973) but drastically reduces the cytotoxicity of even a high concentration (8 mM) of MIS (Fig. 6). In the presence of ASC (5 mM) the same concentration of $O_2$ has much less protective effect (Fig. 6).

Throughout these experiments, some variability between experiments was found for the cytotoxicity of similar concentrations of drug at similar incubation times, as has been found by Hall & Stratford (personal communication). We felt that this might be due to the presence of various labile serum components which might potentiate or decrease the effectiveness of the drug, or even bind to it irreversibly. Therefore we tried various experiments to test for this. It made no difference whether the MIS was dissolved in $H_2O$, balanced salt solution or serum, or whether the mixing solution was sonicated to hasten solubilization, as long as the final drug solution inoculated on to the cells was in complete medium. There was also no effect of mixing the drug at high concentrations and diluting, or mixing the desired concentration outright. However, there was an increase in cytotoxicity when the entire experiment was performed in medium which was either serum-free or contained heat-inactivated serum, particularly in the presence of ASC (Fig. 7). In the presence of heat-inactivated serum one experiment showed toxicity somewhat greater than without serum, and in a second experiment the opposite result was found (data not shown). The differences were so minor, however, that this matter was not pursued.

FIG. 6—Effect of small concentrations of $O_2$ on the cytotoxicity of 8 mM MIS in the presence or absence of 5 mM ASC. □ 8 mM MIS, moderate hypoxia (0·2% $O_2$, MH); ■ 8 mM MIS, extreme hypoxia (EH); ○ 8 mM MIS +5 mM ASC (MH); ● 8 mM MIS +5 mM ASC (EH); ▽ 8 mM MIS+1 mM ASC (EH); △ 2 mM MIS (EH); ▲ 2 mM MIS+5 mM ASC (EH); ⊗ Josephy *et al.* (15 mM MIS+5 mM ASC); × Josephy *et al.* (15 mM MIS). Note that moderate hypoxia (□) almost completely eliminates the very large cytotoxicity seen under extreme hypoxia (■). When 5 mM ASC is added, there is greatly increased killing under EH (●) and MH gives much less protection (○). A single survival point at 4 h showed that 1 mM ASC (▽) was just as effective as 5 mM ASC (●) in potentiating the cytotoxicity of MIS. The arrow on the 4 h point for 8 mM MIS +5 mM ASC (EH) indicates the maximum survival as there were no survivors on these plates. The data for 2 mM MIS (EH) without (△) and with (▲) 5 mM ASC shows that the ascorbate has increased the killing by 2 mM MIS to the level seen by 8 mM MIS without ASC. Data from Josephy *et al.* (1978) for 15 mM MIS (fitted by eye) showed more cytotoxicity in the presence of ASC (⊗) than our 8 mM data (as expected) but less cytotoxicity in the absence of ASC (×) than our 8 mM data. All experimental conditions were tested at 1 h but (△ and □ (100% survival)) were omitted for clarity.

FIG. 7—Effect of serum on cytotoxicity of 2 mM MIS in the presence or absence of 5 mM ASC under conditions of extreme hypoxia at 37°C. ■ 2 mM MIS+serum; □ 2 mM MIS−serum; ▲ 2 mM MIS+5 mM ASC+serum; △ 2 mM MIS+5 mM ASC−serum. The lower values for MIS+ASC (▲, △) are from an independent experiment.

One possible labile serum component is free sulphydryl. In an experiment comparing the cytotoxicity of 5 mM MIS in the presence of ASC and other reducing agents, only ASC potentiated the damage. In fact, 5 mM glutathione, cysteamine-HCl, mercaptoethanol, or cysteine all protected against the toxicity of 2 mM

TABLE.—*Test of several reducing agents on the cytotoxicity of* 2 mM *MIS* (*extreme hypoxia,* 37°C, 6 *h*)

| Drug(s) | Survival (% ± s.e.) |
|---|---|
| Control (No drugs) | 62·0 ± 3·1 |
| 2 mM MIS | 9·8 ± 0·3 |
| 2 mM MIS + 5 mM ASC | 1·3 ± 0·1 |
| 2 mM MIS + 5 mM Glutathione | 55·7 ± 1·5 |
| 2 mM MIS + 5 mM Cysteamine | 69·0 ± 4·0 |
| 2 mM MIS + 5 mM Cysteine | 58·5 ± 3·5 |
| 2 mM MIS + 5 mM Mercaptoethanol | 71·0 ± 3·2 |
| 2 mM MIS + 2·5 mM Na dithionite | 72·0 ± 5·0 |

MIS. Even dithionite (2·5 mM) diminished rather than potentiated the toxicity of 2 mM MIS (Table).

DISCUSSION

The results presented here clearly demonstrate the possible benefits of combined ascorbate-sensitizer with respect to hypoxic-cell killing *in vitro* (Figs. 2, 3, 6, 7). The effects of high doses of ASC *in vivo*, however, are more difficult to predict. For example, ASC could on the one hand increase the cytotoxicity of radio-sensitizing drugs, but on the other hand increase the fraction of hypoxic cells, through auto-oxidation processes (Fig. 4). This, in fact, may be the cause of the increased cytotoxicity seen with ASC–MIS at a gas-phase $O_2$ partial-pressure of 2000 pts/$10^6$ (Fig. 6); in other words, the auto-oxidation of ascorbate decreases the concentration of $O_2$ in the medium below the gas-phase value. However, *in vivo*, the rate of cellular $O_2$ consumption would be about $5{\cdot}4 \times 10^{-2}$ mol/h [assuming a cell concentration of $5 \times 10^8$/g and an $O_2$ consumption rate of $5 \times 10^{-17}$ mol/cell/sec (Koch & Biaglow, 1978*b*)]. In contrast, the auto-oxidation rate of 2 mM ASC+2 mM MIS in BME is about 500× less than this (Fig. 4b). Thus, the auto-oxidation of vitamin C would not be expected to change the overall $O_2$ concentration significantly, except perhaps in local areas where the $O_2$ concentration was already very low.

The contrasting effects of ascorbate and SH-containing compounds warrant considerable further investigation. In particular, combinations of sensitizer, ASC, and RSH should be tested in the cytotoxicity assay. This is essential, to show which effect predominates (enhancement of toxicity by ASC, reduction of toxicity by RSH) and whether there is a chemical competition between the effects. A significant concentration of RSH or RSSR certainly exists *in vivo*. One published report has shown that cysteamine protects against both the radiosensitizing and cytotoxic effects at about the same concentration (Hall *et al.*, 1977). Thus the fact that significant radiosensitization has been shown for hypoxic cells *in vivo* already suggests that free SH may not be high enough to be a significant problem in the use of agents like MIS. In addition it is necessary to identify the precise concentrations of ASC or RSH necessary to achieve full potentiation or decrease in cytotoxicity. Preliminary evidence would indicate that 1 mM ASC is just as effective as 5 mM (Fig. 6).

This points to important differences between our results and those of Josephy *et al.* (1978). Those investigators found a greater potentiation by ascorbate of the cytotoxicity of MIS than we did, and in addition, found a significant dependence of the cytotoxicity on vitamin C concentration. They also found potentiation of the cytotoxicity of MIS by glutathione. We believe that these discrepancies can be explained by incomplete $O_2$ removal from their test system. This would explain the relatively smaller amount of toxicity seen for 5 and 15 mM MIS alone (compared with other reports—Hall *et al*, 1977; Stratford & Adams, 1977; Mohindra & Rauth, 1976; this paper) because of the protective effect of $O_2$ (Fig. 6). Thus the addition of ASC would not only increase the cytotoxicity, but decrease the $O_2$ concentration (Fig. 6 and see previous discussion). Finally, the small amount of potentiation reported by Josephy *et al.* for glutathione could also be caused by auto-oxidation of glutathione, leading to reduced $O_2$ levels. The protection by RSH of the cytotoxicity of MIS observed in our results agrees completely with a previous report by Hall *et al.* (1977), who found that cysteamine not only nullified the cytotoxicity of MIS, but also cancelled its radiosensitizing properties. Our results show that ASC potentiated the cytotoxicity and did not affect the radiosensitizing properties of MIS (Fig. 5).

A major problem in the comparison of the cytotoxic effects of misonidazole in different laboratories involves traces of $O_2$, and components of serum which are as yet unidentified. Thus, Mohindra & Rauth (1976) and Stratford (1978) have found that relatively small concentrations of $O_2$ can greatly reduce the cytotoxic effects of radiosensitizers (Fig. 6). Quantitative assessments of this reduction as a function of $O_2$ concentration have not yet been made, and may very well depend on the electron affinity of the drug and the presence or absence of compounds like RSH in the medium. Even greater problems may occur because of the unpredictable effects of serum. Thus, Stratford & Gray (1978) found potentiation of cytotoxicity by serum whereas we have found reduction of cytotoxicity by serum. In retrospect these differences might be expected, since the action of reducing agents like ASC and RSH are opposite. However, it is of interest that the survival curves (2 mM MIS, extreme hypoxia) for the V79 cells used by Stratford & Gray (1978) and for the V79 cells used in our present experiments are very similar when no serum is present (compare Fig. 3 of Stratford paper with Fig. 7 of this paper). This important internal agreement suggests that the serum effects are real and that the chemicals responsible should be identified.

Finally, at the chemical level, we still do not understand the role of ASC in the killing of hypoxic cells by agents like MIS and indeed the toxic species itself has not been identified. If the toxic species is the same for both MIS and (MIS+ASC), ASC is probably acting by increasing the reduction (presumably metabolic) of MIS to a more harmful species. Alternatively, the increased toxicity may be caused by an oxidation product of vitamin C (*e.g.* semi-quinone), whose mode of action may be similar to that of dehydroascorbic acid (Koch & Biaglow, 1978*a*). Our current experiments are aimed at trying to elucidate the killing mechanisms for these drugs.

This work was supported by the Ontario Cancer Treatment & Research Foundation, London Clinic. The authors thank Dr. C. Smithen (Roche Products) for the gift of misonidazole.

REFERENCES

ADAMS, G. E. & COOKE, M. S. (1969) Electron affinic sensitization. 1: A structural basis for chemical radiosensitizers in bacteria. *Int. J. Radiat. Biol.*, **15**, 457.

BIAGLOW, J. E., JACOBSON, B. & KOCH, C. (1976) The catalytic effect of the carcinogen "4-nitroquinoline-N-oxide" on the oxidation of vitamin C. *Biochem. Biophys. Res. Commun.*, **70**, 1316.

BIAGLOW, J. E., JACOBSON, B., VARNES, M. & KOCH, C. (1978) The oxidation of ascorbate by electron affinic drugs and carcinogens. *Photochem. Photobiol.*, (in press).

BROWN, J. M. (1977) Cytotoxic effects of the hypoxic cell radiosensitizer Ro-07-0582 to tumor cells *in vivo*. *Radiat. Res.*, **72**, 469.

CHAPMAN, J. D., REUVERS, A. P., BORSA, J., HENDERSON, J. A. & MIGLIORE, R. D. (1974) Nitro-heterocyclic drugs as selective radiosensitizers of hypoxic mammalian cells. *Cancer Chemother. Rep.*, **58**, 559.

ELKIND, M. M., SWAIN, R. W., ALESCIO, T., SUTTON, H. & MOSES, W. B. (1965) Oxygen, nitrogen, recovery and radiation therapy. In: *Symp. Fundam. Cancer Res.*, Baltimore: Williams and Wilkins. p. 442.

HALL, E. J., ASTOR, M., GEARD, C. & BIAGLOW, J. (1977) Cytotoxicity of Ro-7-0582; Enhancement by hyperthermia and protection by cysteamine. *Br. J. Cancer*, **35**, 809.

JOSEPHY, P. D., PALCIC, B. & SKARSGARD, L. D. (1978) Ascorbate-enhanced cytotoxicity of misonidazole. *Nature*, **271**, 370.

KOCH, C. J. & BIAGLOW, J. E. (1978*a*) Radiation sensitivity modification and metabolic effects of dehydroascorbate and ascorbate in mammalian cells. *J. Cell. Physiol.*, **94**, 299.

KOCH, C. J. & BIAGLOW, J. E. (1978*b*) Respiration of mammalian cells at low concentrations of oxygen: I. Effect of hypoxic-cell radiosensitizing drugs. *Br. J. Cancer*, **37**, (Suppl. III), 163.

KOCH, C. J. & PAINTER, R. B. (1975) The effect of extreme hypoxia on the repair of DNA single-strand breaks in mammalian cells. *Radiat. Res.*, **64**, 256.

KOCH, C. J., KRUUV, J. & FREY, H. E. (1973) Variation in radiation response of mammalian cells as a function of oxygen tension. *Radiat. Res.*, **53**, 33.

KOCH, C. J., MENESES, J. & HARRIS, J. W. (1977) The effect of extreme hypoxia and glucose on repair of potentially lethal and sublethal radiation damage by mammalian cells. *Radiat. Res.*, **70**, 542.

MITCHELL, J. S. & MARRIAN, D. H. (1965) Radiosensitization of cells by a derivative of 2-methyl-1,4 napthoquinone. *Biochemistry of Quinones.* Ed. R. A. Morton, London: Academic Press. p. 503.

MOHINDRA, J. E. & RAUTH, A. M. (1976) Increased killing by metronidazole and nitrofurazone of hypoxic compared to aerobic mammalian cells. *Cancer Res.*, **36**, 930.

PETERKOFSKY, G. & PRATHER, W. (1977) Cytotoxicity of ascorbate and other reducing agents towards cultured fibroblasts as a result of hydrogen peroxide production. *J. Cell. Physiol.*, **90**, 61.

RAUTH, A. M., CHIN, J., MARCHOW, L. & PACIGA, J. (1978) Testing of hypoxic cell radiosensitizers *In Vivo*. *Br. J. Cancer*, **37**, (Suppl. III), 202.

SCHNEIDER, E. L., STANBRIDGE, E. J. & EPSTEIN, C. J. (1974) Incorporation of $^3H$-uridine and

$^{3}$H-uracil into RNA: a simple technique for the detection of mycoplasma contamination of cultured cells. *Exp. Cell Res.*, **84**, 311.

SRIDHAR, R. & SUTHERLAND, R. S. (1977) Hyperthermic potentiation of cytotoxicity of Ro-07-0582 in multicell spheroids. *Int. J. Radiat. Oncol. Biol. Phys.*, **2**, 531.

SRIDHAR, R., KOCH, C. & SUTHERLAND, R. (1976) Cytotoxicity of two nitroimidazole radiosensitizers in an *in vitro* tumor model. *Int. J. Radiat. Oncol. Biol. Phys.*, **1**, 1149.

STRATFORD, I. J. & ADAMS, G. E. (1977) Effect of hyperthermia on differential cytotoxicity of hypoxic cell radiosensitizer Ro-07-0582, on mammalian cells *in vitro*. *Br. J. Cancer*, **35**, 307.

STRATFORD, I. J. & GRAY, P. (1978) Some factors affecting the specific toxicity of misonidazole towards hypoxic mammalian cells. *Br. J. Cancer*, **37**, (Suppl. III), 129.

STRATFORD, I. J. (1978) Split dose cytotoxic experiments with misonidazole. *Br. J. Cancer*, **38**, 130.

SUTHERLAND, R. M. (1974) Selective chemotherapy of noncycling cells in an *in vitro* tumor model. *Cancer Res.*, **34**, 3501.